# Novel Gold Dendritic Nanoforests Combined with Titanium Nitride for Visible-Light-Enhanced Chemical Degradation

**DOI:** 10.3390/nano8050282

**Published:** 2018-04-26

**Authors:** Ming-Hua Shiao, Chun-Ting Lin, Jian-Jia Zeng, Yung-Sheng Lin

**Affiliations:** 1 Instrument Technology Research Center, National Applied Research Laboratories, Hsinchu 300, Taiwan; mhshiao@narlabs.org.tw (M.-H.S.); chunting.pineapple.lin@gmail.com (C.-T.L.); 2 Department of Chemical Engineering, National United University, Miaoli 360, Taiwan; l82178253@gmail.com

**Keywords:** photocatalyst, degradation, gold, titanium nitride

## Abstract

In this study, gold dendritic nanoforests (Au DNFs)-titanium nitride (TiN) composite was firstly proposed for visible-light photodegradation of pollutants. A high-power impulse magnetron sputtering system was used to coat TiN films on silicon wafers, and a fluoride-assisted galvanic replacement reaction was applied to deposit Au DNFs on TiN/Si substrates. Scanning electron microscope images and X-ray diffraction patterns of TiN/Si, Au DNFs/Si, and Au DNFs/TiN/Si samples verified that this synthesis process was accurately controlled. The average reflectance of Au DNFs/Si and Au DNFs/TiN/Si considerably declined to approximately 10%, because the broadband localized surface plasmon resonances of Au DNFs cause broadband absorbance and low reflectance. In photocatalytic performance, 90.66 ± 1.41% 4-nitrophenol was successfully degraded in 180 min by Au DNFs/TiN/Si under visible-light irradiation. Therefore, Au DNFs/TiN/Si has the chance to be a visible-light photocatalyst for photodegradation of pollutants.

## 1. Introduction

Titanium dioxide (TiO_2_) is the most widely used photocatalyst in the photodegradation of pollutants because of its strong oxidizing abilities, chemical stability, nontoxicity, and low cost [1]. Although TiO_2_ is widely used as a benchmark for other photocatalysts, TiO_2_ only absorbs the ultraviolet (UV) region of the solar spectrum; this limited absorption is an obstacle to the wide application of TiO_2_ for the photodegradation of pollutants [2]. Therefore, various photosensitized degradation processes have attracted considerable attention because they can harvest maximal solar energy by utilizing visible light to degrade pollutants [2]. Introducing nanosized metal materials [3,4,5,6,7] or a doping N element [8] to reduce the band gap energy can extend the activating spectrum of TiO_2_ from UV to the visible spectrum and enhance performance.

Titanium nitride (TiN) is an extremely hard ceramic material with well-known thermal, chemical, and mechanical stability properties. TiN is applied as a thin film coating to harden and protect cutting and sliding surfaces of industrial machine tools. TiN has particular optical and metallic properties and possesses potentially resonant plasmon characteristics in the visible spectrum [9,10,11]. A study demonstrated that TiN can exhibit electromagnetic field enhancements comparable to those of gold (Au) nanostructures [12]. TiN exhibited higher electrical conductivity and optical absorption in long wavelength range than Ti [13]. Studies of depositing silver [14,15,16] or copper [17] on TiN substrates for applications have been reported. However, few studies have been published regarding gold nanostructure/TiN composite materials for applications. 

Gold nanoparticles and nanostructures exhibit characteristic optical properties because of localized surface plasmon resonance (LSPR), which are dependent on the sizes and shapes of nanomaterials [18]. Therefore, gold nanoparticles and nanostructures with different sizes have different characteristic LSPR wavelengths. Gold dendritic nanoforests (Au DNFs) have attracted considerable attention due to their noteworthy specific surface area and strong LSPR enhancement for wide wavelengths [19]. However, the synergetic effects of Au DNFs combined with TiN on photodegradation have not been reported. In this study, Au DNF/TiN/Si composite samples were applied to photocatalytically degrade organic dyes under visible-light illumination.

## 2. Materials and Methods

### 2.1. Sample Preparation

TiN films were deposited on silicon wafers by physical vapor deposition with a high-power impulse magnetron sputtering system (HIPIMS) [20]. In brief, a 4-inch-diameter titanium target was mounted on one cathode and a pulse generator with a frequency of 20 kHz was applied. The pressure of the deposition chamber was pumped down to less than 8 × 10^−6^ Torr by using a cryopump. Before the deposition of the TiN layer, a Ti layer was deposited on the silicon wafer to connect Si and TiN. The parameters used in the chamber to prepare Ti and TiN layers are listed in Table 1. In this study, the thicknesses of Ti and TiN were 50 and 300 nm, respectively.

The synthesis of Au DNFs basically follows a fluoride-assisted galvanic replacement reaction (FAGRR) [19] that is widely used to deposit metal nanoparticles on Si substrate. The reactions are generally agreed as follow:

Anode: Si + 6 F^−^ → SiF_6_^2−^ + 4 e^−^

Cathode: AuCl_4_^−^ + 3 e^−^ → Au + 4 Cl^−^

In the case of TiN substrate, the anodic reaction proceeds as follow [21]:

Anode: TiN + 3HF_2_^−^ → TiF_6_^2^^−^ + NH_3_ + e^−^

An *n*-type silicon wafer (2 × 2 cm) was dipped in a mixture of 10 mM chloroauric acid (HAuCl_4_) and buffered oxide etchant solution containing 11.3% NH_4_F and 2.3% HF for 3 min in a typical manner. Then, the sample was washed with deionized water, dried using an N_2_ spray, and incubated at 120 °C for 5 min to obtain an Au DNFs/Si sample. The Au DNFs/TiN/Si synthesis procedure was the same except that it used a TiN/Si substrate.

### 2.2. Photodegradation

The photodegradation experiment was conducted in a 250-mL beaker. A solar simulator (class B; λ > 400 nm; 150 W; Sadhu Design Corporation, Hsinchu, Taiwan) was employed as the light source for the photodegradation experiment. The tested sample (2 cm × 2 cm) was immersed in 10 mL 4-nitrophenol solution containing 2.5 × 10^−5^ M 4-nitrophenol and 0.125 M NaBH_4_, and the illumination power density was controlled at 70 mW/cm^2^. The concentration of 4-nitrophenol was examined according to an absorption calibration curve at its maximum absorption wavelength. The degradation process was monitored every 30 min for 3 h. All of the experiments were conducted in the ambient conditions.

## 3. Results and Discussion

### 3.1. Morphology and Elemental Analysis

**Figure 1:**
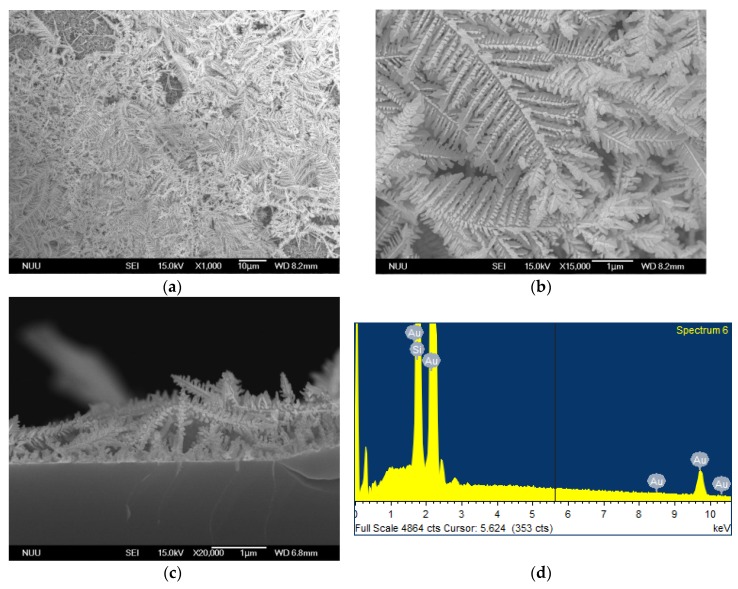
Scanning electron microscope (SEM) examination of Au DNFs/Si. (**a**,**b**) top view; (**c**) side view; (**d**) Energy-dispersive X-ray spectroscopy (EDS) spectrum.

Figure 1 shows scanning electron microscope (SEM) images and the corresponding energy-dispersive X-ray spectroscopy (EDS) spectrum of Au nanostructures on a silicon wafer prepared by a FAGRR. Results indicated that Au DNFs contained multiple levels of branches with features measuring 30–50 nm (Figure 1a,b). The side view picture (Figure 1c) indicated that evenly distributed Au DNFs were highly porous and the average thickness of free-standing Au DNFs was approximately 800 nm on the Si wafer. In an element analysis, only Au and Si signals were found in the Au DNFs/Si sample (Figure 1d), and the results reflected the absence of other byproducts from the FAGRR [19].

**Figure 2:**
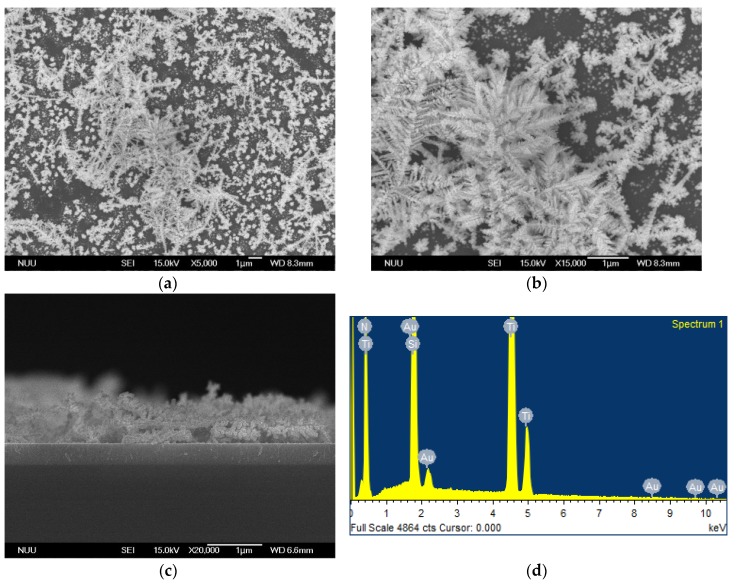
SEM examination of Au DNFs/TiN/Si. (**a**,**b**) top view; (**c**) side view; (**d**) EDS spectrum.

Figure 2 presents SEM images and an EDS spectrum of Au nanostructures on a TiN/Si substrate fabricated by a FAGRR. Results indicated that leaf-like Au DNFs were loosely distributed on TiN/Si (Figure 2a,b). The Au DNFs/TiN/Si sample had a lower density of Au DNFs than did the Au DNFs/Si sample. The thickness of the Au DNFs was only approximately 650 nm (Figure 2c). Only Au, Ti, N, and Si signals were evident from the Au DNFs/TiN/Si sample (Figure 2d), corresponding to the result for the Au DNFs/Si sample.

**Figure 3:**
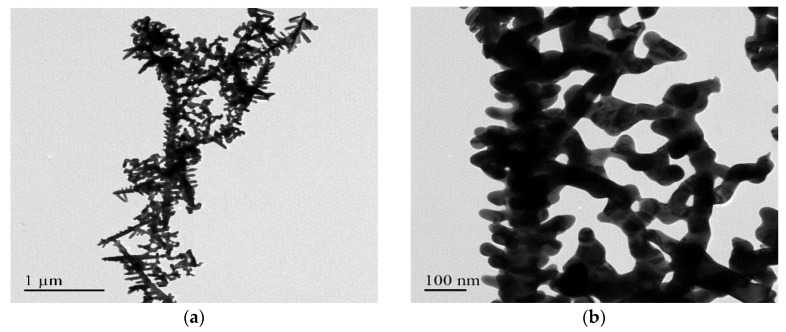

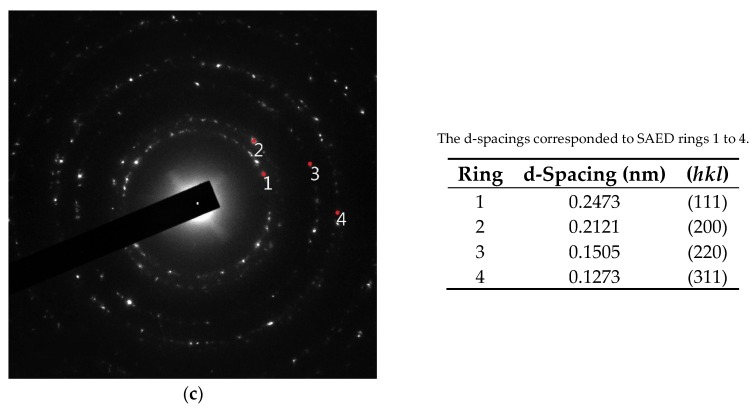
Transmission electron microscope (TEM) examination of Au DNFs deposited on Si substrate. (**a**) Low-magnification; (**b**) High-magnification; (**c**) corresponding selected area electron diffraction (SAED) pattern of (**b**).

**Figure 4:**
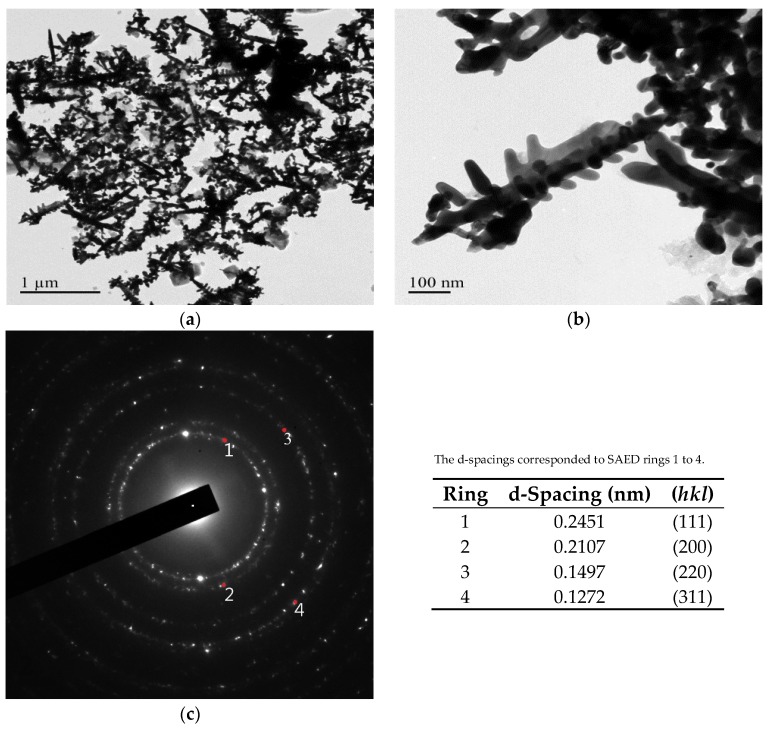
TEM examination of Au DNFs deposited on TiN/Si substrate. (**a**) Low-magnification; (**b**) High-magnification; (**c**) corresponding SAED pattern of (**b**).

Figure 3 and Figure 4 present transmission electron microscope (TEM) images and selected area electron diffraction (SAED) patterns of Au DNFs deposited on Si and TiN/Si substrates, respectively. To get clear TEM bright field images and diffraction information of Au DNFs, the deposition time of Au DNFs was controlled for 1 min. The results indicate bright field images of Au DNFs deposited on both Si and TiN/Si substrates, and show the thickness of Au branches of less than 50 nm, which is suitable for TEM observations. It can be obviously observed that the trunks in the Au DNFs deposited on a single crystal of Si wafer substrate in Figure 3a are straighter and longer than those deposited on polycrystalline TiN substrate in Figure 4a. The SAED patterns reveal that the d-spacings of (111), (200), (220), and (311) planes corresponded to each of the diffraction rings in Figure 3c and Figure 4c.

**Figure 5:**
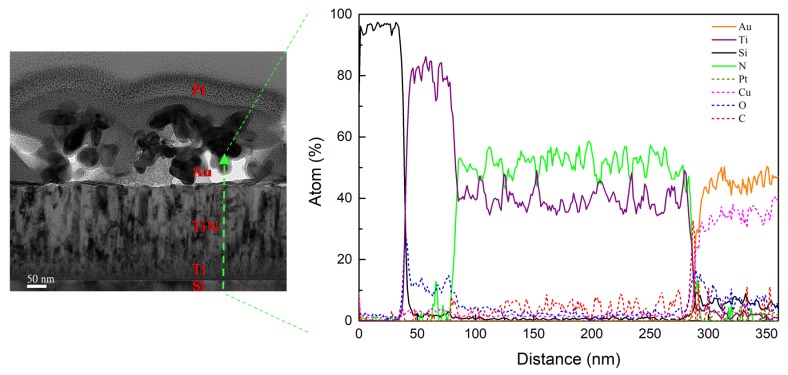
TEM elemental analysis of Au DNFs/TiN/Si sample.

Figure 5 further shows the TEM cross-section image of Au DNFs on the TiN/Si substrate, and the EDS line scan from Au DNFs cross the TiN layer to Si substrate. The microstructure of Au DNFs and columnar structure of TiN coating can be observed. The EDS line scan shows the measured intensities of Au, Ti, N, and Si elements corresponded to the procedures we applied on the silicon wafer in sequence. The intensities of C, O, Pt, and Cu were the background signals. Except background signals, there were almost only Ti signals at the interface of the TiN and Si substrate because a 50 nm Ti layer was deposited between the TiN layer and Si substrate (Table 1).

**Figure 6:**
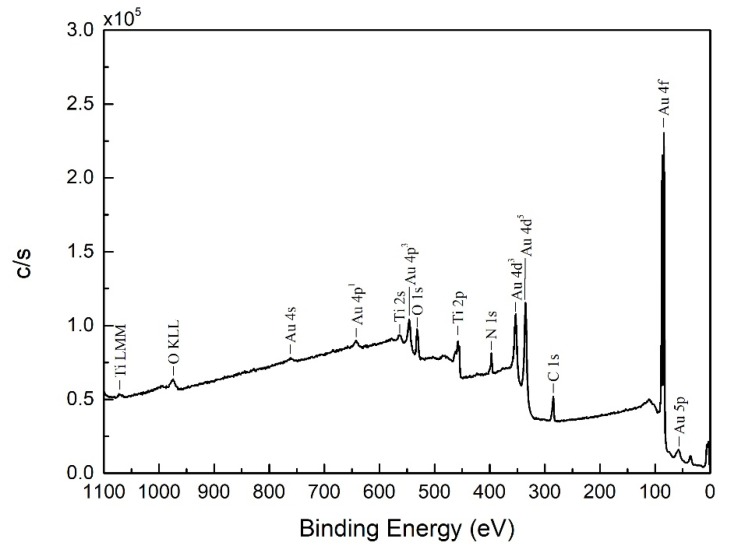
X-ray photoelectron spectroscopy (XPS) examination of Au DNFs deposited on TiN/Si substrate.

Figure 6 shows the X-ray photoelectron spectroscopy (XPS) pattern of Au DNFs deposited on the TiN/Si substrate. The data was collected from a top plan of Au DNFs/TiN/Si sample. Results indicated that only Au, Ti, N, C, and O signals were evident. The atomic percentages were 34.2%, 19.3%, 18.9%, 15.6%, and 12.0% for C 1s, O 1s, Au 4f, N 1s, and Ti 2p, respectively. Among these elements, C and O were the background signals. Therefore, this result reflected the good control in preparing the Au DNFs/TiN/Si sample.

### 3.2. Crystalline

**Figure 7:**
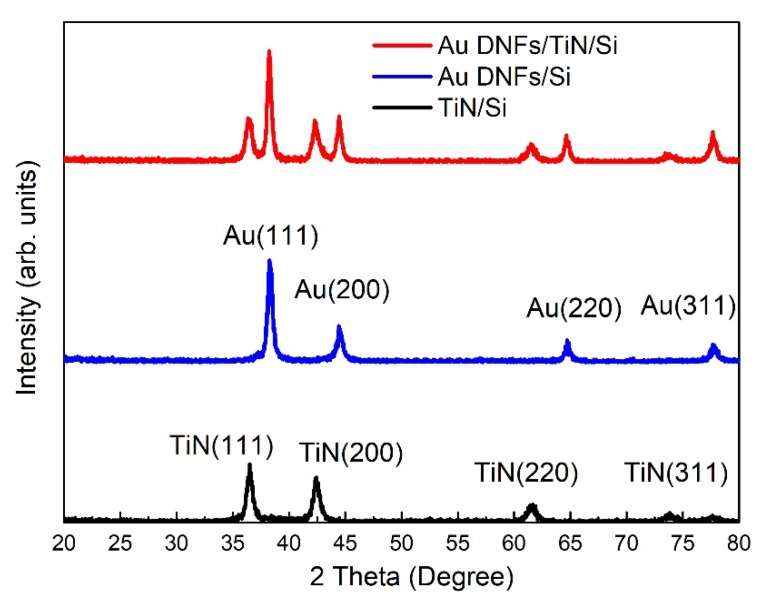
X-ray diffraction (XRD) patterns of the TiN/Si, Au DNFs/Si, and Au DNFs/TiN/Si.

Figure 7 shows the X-ray diffraction (XRD) pattern of Au samples deposited on a Si surface. Results indicated that Au has polycrystalline characteristics. Peaks of Au(111), Au(200), Au(220), and Au(311) were found in both Au DNFs/Si and Au DNFs/TiN/Si, which agreed with the information regarding Au found in JCPDS 04-0784. The growth of Au in this manufacturing process has a strong preferential (111) orientation, as has been reported in previous articles [22,23]. The intensity ratios of the Au(111)/Au(200) and Au(111)/Au(220) peaks amounted to 2.82 and 4.64 for Au DNFs/Si, 2.42 and 4.12 for Au DNFs/TiN/Si. The XRD results of Au DNFs agree with the SAED results in Figure 3c and Figure 4c. TiN(111), TiN(200), TiN(220), and TiN(311) were shown in both TiN/Si and Au DNFs/TiN/Si, which corresponded to the TiN information in JCPDS38-1420. The preferential orientation of TiN was also (111). The intensity ratios of the TiN(111)/TiN(200) and TiN(111)/TiN(220) peaks amounted to 1.30 and 3.20 for TiN/Si, 1.06 and 2.43 for Au DNFs/TiN/Si.

### 3.3. Reflectance

**Figure 8:**
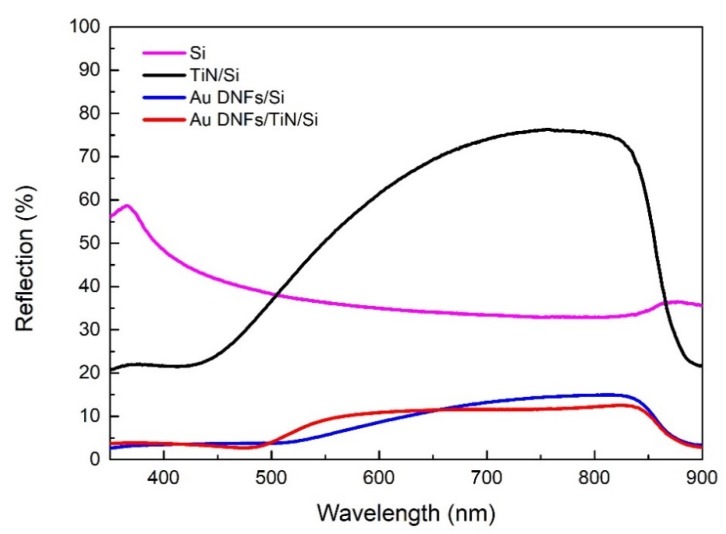
Reflectance spectra of different samples.

Figure 8 shows that a Si wafer had an average reflectance of approximately 40%. The Au DNFs/Si or Au DNFs/TiN/Si composites exhibited multiple scattering of incident light between the DNFs, which enhanced light trapping [24]. Therefore, Au DNFs/Si or Au DNFs/TiN/Si exhibited antireflection characteristics with an average reflectance of approximately 10%; in particular, the reflectance in the low wavelength region (λ < 500 nm) decreased considerably. Au DNFs with random branches had different sizes, shapes, and gap sizes and thus had various LSPR bands. Broadband LSPR causes broadband absorbance and low reflectance. A similar broadband behavior for the UV-visible spectrum has also been reported for Si-supported Au nanostructures [19].

The reflectance spectrum of Au DNFs/TiN/Si followed that of Au DNFs /Si. That indicates the Au nanostructures grown on the TiN layer dominate the reflectance process. The difference between Au DNFs/TiN/Si and Au DNFs/Si may be attributed to the synergetic effect of LSPR between the gold nanostructures and TiN film. Although the thickness and density of Au DNFs on substrates were both smaller for Au DNFs/TiN/Si than for Au DNFs/Si, the average reflectance did not vary much due to the addition of TiN, which had resonant plasmon characteristics in the visible spectrum [9,10].

### 3.4. Photodegradation

**Figure 9:**
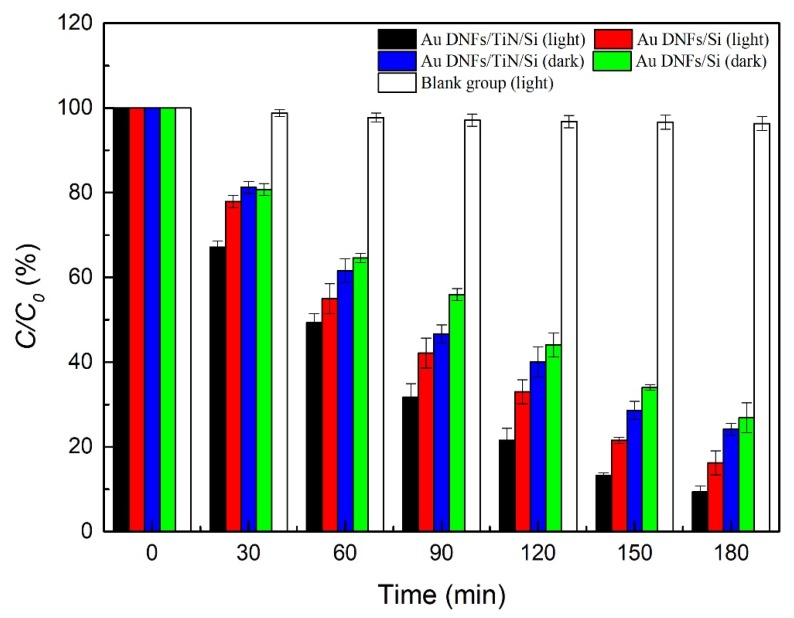
Photodegradation of 4-nitrophenol in 180 min.

Figure 9 presents photodegradation of 4-nitrophenol. Two samples, Au DNFs/Si, and Au DNFs/TiN/Si, were tested in the presence of illumination or in dark environments. Agreeing with previous reports [25,26,27], results indicated 4-nitrophenol broke down notably in appearance, and concentrations of 4-nitrophenol decreased with time. In 180 min, for the Au DNFs/Si, and Au DNFs/TiN/Si, the concentrations of 4-nitrophenol were 16.25 ± 2.83% and 9.33 ± 1.41%, respectively, in the presence of illumination, and 26.82 ± 3.54% and 24.16 ± 1.41%, respectively, in the dark. The concentrations in illumination were smaller than dark environments, and therefore illumination showed more photodegradation of 4-nitrophenol than dark. As for the effect of substrate, Au DNFs/TiN/Si had more photodegradation than Au DNFs/Si.

**Figure 10:**
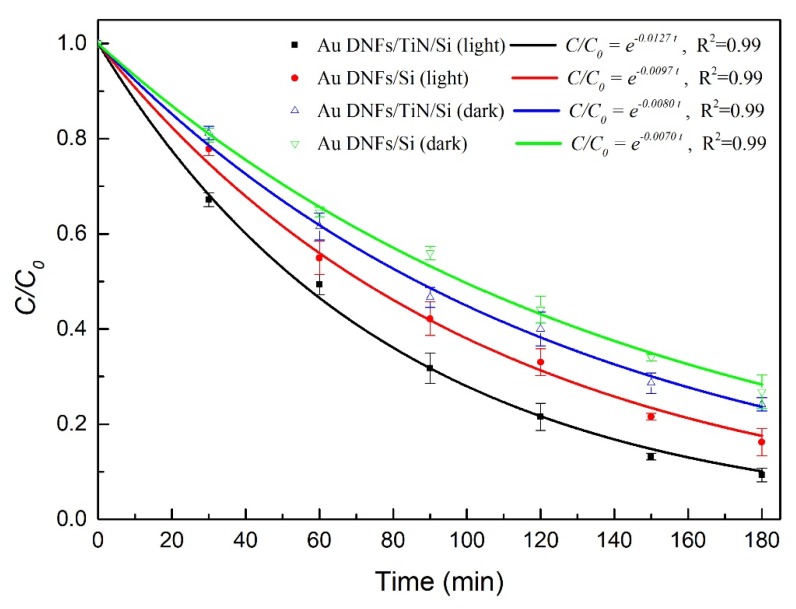
Curve fitting of 4-nitrophenol photodegradation in 180 min.

Figure 10 is the curve fitting by the first-order reaction and Table 2 summarizes the reaction rate constant in the photodegradation reaction described by an equation

*C/C*_0_ = *e^−kt^* (1)

where *C/C*_0_ is the concentration fraction, *t* is the reaction time, and *k* is the reaction rate constant. The optimal degradation happened on the Au DNFs/TiN/Si composite in the presence of illumination.

**Figure 11:**
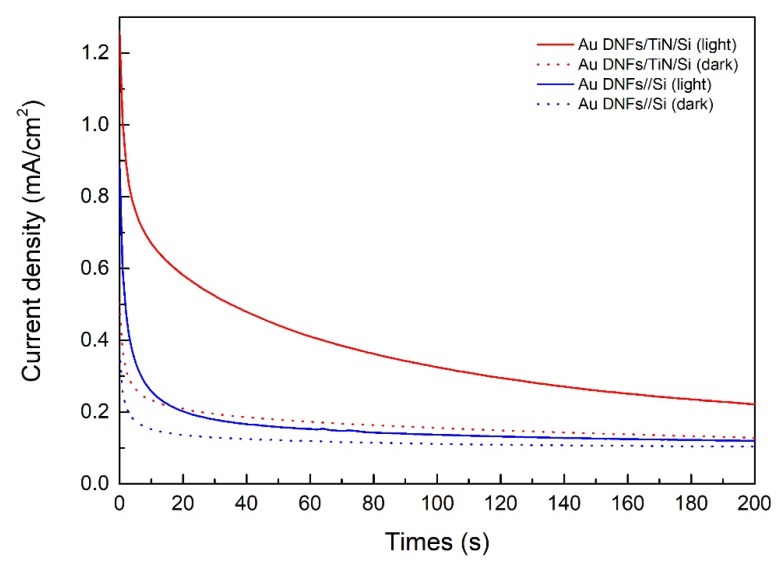
Chronoamperometry test of 4-nitrophenol solution.

Figure 11 is the chronoamperometry test of 500 mL 2.5 × 10^−5^ M 4-nitrophenol solution with 0.5 M NaOH. Results indicated that photocatalytical reaction of 4-nitrophenol was enhanced under illumination. Regardless of the samples, the current density under illumination was higher than that in the dark. In addition, Au DNFs/TiN/Si had a higher current density than Au DNFs/Si, corresponding to the photodegradation result in Figure 9.

**Figure 12:**
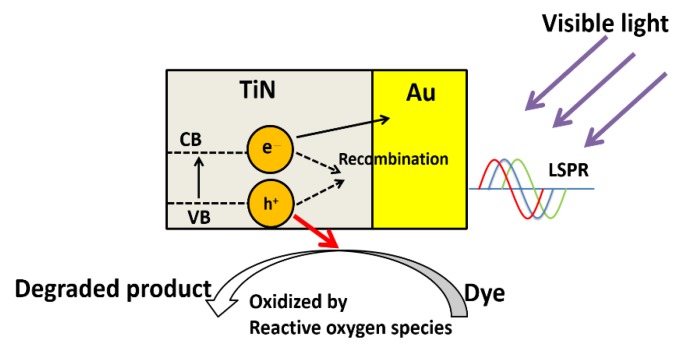
The schematic diagram of visible-light induced degradation on Au-TiN composites.

The visible-light enhanced degradation could be attributed to the synergetic effect between Au DNFs and the TiN layer. Figure 12 shows the schematic diagram of visible-light induced degradation on Au-TiN composites. The Au DNFs/Si and Au DNFs/TiN/Si composites can exhibit multiple scattering of incident light between the DNFs and enrich light trapping [24]. Besides, the enhanced efficiency of 4-nitrophenol degradation for Au DNFs/Si and Au DNFs/TiN/Si under illumination is attributed to the broadband LSPR generated on Au nanoforests. The Au nanoforests provide multiple sizes and various directions to harvest the broadband illumination. A previous publication reported the visible light enhanced methanol oxidation by silicon-based gold nanodendrites [19]. It is also widely accepted that gold nanostructures can serve as the photocatalyst due to the LSPR generated under illumination [19,27,28,29]. The enhanced performance of gold nanostructures for LSPR applications are reported [30,31].

The photodegradation results (Table 2) show that the efficiency of photodegradation is enhanced on Au DNFs/TiN/Si comparing to Au DNFs/Si. We attributed the results to the plasmonic effect of TiN supported Au DNFs and band modification of TiN by Au nanostructures. The combination of Au DNFs and TiN can reduce the band gap of TiN to promote electrons from the valence band of TiN into the conduction band [32]. As generally agreed in the metal-semiconductor interface, the gold nanostructures can serve as the sink for electrons and increase the lifetime of the photogenerated electrons and holes under illumination. The longer lifetimes of the photoexcited electron-hole pairs favor the formation of reactive oxygen species, such as superoxides (O_2_^−^), hydroxyl radicals (HO·), and etc. [33]. The highly reactive oxygen species can react with and destroy organic dyes by a fast ring-opening process and a complete mineralization of chromophores [32,34].

## 4. Conclusions

In this study, Au DNFs was firstly deposited on the TiN for photocatalysis. Hierarchical Au DNFs with multiple branch sizes were decorated on the surface of TiN/Si by FAGRR for the photodegradation of organic dyes under visible-light illumination. In photodegradation tests, Au DNFs/TiN/Si successfully degraded approximately 90.66 ± 1.41% 4-nitrophenol using visible light within 180 min. The enhancement of photodegradation could be attributed to the coupling effect between the LSPR and band modification of TiN. The Au DNFs/TiN/Si composites proposed in this paper show a potential to be used in visible-light induced photodegradation. Additionally, the facile electroless deposition process introduced in this paper provides a new approach in preparing metal-TiN composites.

## Figures and Tables

**Table 1:** High-power impulse magnetron sputtering system (HIPIMS) parameters for deposition of Ti and TiN layers.

Layer	DC Power (W)	Impulse Period (µs)	Ar Flow Rate (sccm)	N_2_ Flow Rate (sccm)
Ti	250	90	20	-
TiN	300	1000	30	1.5

**Table 2:** The reaction rate constant in the photodegradation reaction.

Sample	Reaction Rate Constant (×10^−3^, 1/min)
Au DNFs/Si (dark)	7.0
Au DNFs/TiN/Si (dark)	8.0
Au DNFs/Si (light)	9.7
Au DNFs/TiN/Si (light)	12.7
